# Automated measurement: The need for a more objective view of the speech and language of autistic children

**DOI:** 10.3389/fnhum.2023.1124273

**Published:** 2023-04-06

**Authors:** Eraine Leland, Regina M. Fasano, Jacquelyn M. Moffitt, Celia Romero, Catalina Cepero, Daniel S. Messinger, Lynn K. Perry

**Affiliations:** ^1^Department of Psychology, University of Miami, Coral Gables, FL, United States; ^2^Department of Pediatrics, University of Miami, Miami, FL, United States; ^3^Department of Electrical and Computational Engineering, University of Miami, Coral Gables, FL, United States; ^4^Department of Music Engineering, University of Miami, Coral Gables, FL, United States

**Keywords:** autism, ASD, speech and language, language development, automated measures, LENA

## Introduction

Autistic^1^children's language often develops differently than that of neurotypical (NT) children. Standardized assessments of language are useful in educational and clinical practice but are unable to fully capture a child's day-to-day speech and language. This opinion paper discusses how automated measurement allows researchers to quickly collect more objective data and measure multiple aspects of language and interactions in real-world contexts. Measurement of vocalizations *via* the Language ENvironment Analysis (LENA) system is a common thread throughout the studies included, as it is widely used in studies on speech and language. However, we also discuss several studies that have used other automated measures in conjunction with LENA to give a richer picture of autistic children's language in context. We argue that use of such emerging measurement technologies provides insights into children's language use in day-to-day life and helps us better understand group differences between autistic and NT children. We also detail implications for clinical practice and suggest future directions for automated measurement in autism research.

## Traditional approaches for studying speech and language in autism

Rich early language environments are linked to better later language abilities (Gilkerson et al., 2018). They are especially important for children with increased likelihood of language delay, including children with an elevated likelihood of autism (Romeo et al., 2022). Language abilities are usually assessed in educational and clinical settings *via* standardized assessments. Assessments provide information about children's strengths and challenges compared to a normative sample. As such, minimally verbal children's results may be inadequate due to floor effects (Chenausky et al., 2022). Missing from standardized assessments is an understanding of children's speech and language in the context of day-to-day life. Some children struggle to engage during assessments, while others who have difficulty engaging in social interactions might be *more* engaged during the highly structured assessment. Therefore, assessment scores may not be particularly representative of the language typically used by autistic children.

Historically, speech and language were measured in context by observational coding of language at home, in the clinic, or in preschools. Whether done live or from video, hand-coding speech is time and labor intensive. This task is even more daunting when coding developing/unclear speech or fine-grained components of speech, such as phonemic complexity and pitch, or temporal patterns of interactions. Additionally, hand-coding is inherently subjective. Furthermore, observational coding requires focusing on the interactions of a single individual or dyad, ignoring other dynamic language experiences and interactions occurring around the child. In contrast, automated measurements facilitate the objective acquisition of large amounts of data from naturalistic and clinical environments in significantly less time. Such technologies can measure multiple levels of speech from multiple people at once in real-world contexts, enabling researchers to more readily assess how linguistic behaviors unfold. Analysis of such large, multi-dimensional data can also extend our understanding of associations between linguistic behaviors and autism symptoms. Here, we consider examples from recent salient papers relevant to our work in the field demonstrating how automated speech and language measures have been used in diverse real-world contexts and yielded new insights into ASD symptoms and developmental outcomes.

## Automated measures of vocalizations and social interactions

### Vocalizations and conversational turn count

One of the most frequently used automated measurers of vocalizations is the Language ENvironment Analysis (LENA) system, a lightweight child-worn recording device and diarization software. LENA software estimates the number of recorded target child vocalizations, other child (e.g., peers and siblings) vocalizations, and adult word counts (AWC). LENA has been used in clinic, home, and school settings to capture the language of autistic and NT children. One key language experience captured by LENA is conversational turn-taking (Donnelly and Kidd, 2021), which occurs when a child vocalization and an adult vocalization occur within 5 s of each other. LENA-measured conversational turn-counts (CTC) have been found to predict changes in cortical regions associated with language production and processing and executive function development (Romeo et al., 2021). Home LENA recordings show that although AWC is similar in autistic and NT preschoolers' homes, autistic children engage in fewer CTCs than NT children (Warren et al., 2010).

LENA also allows comparison of individual language experiences of autistic and NT students in the same classroom. Much like at home, autistic preschoolers engaged in fewer CTCs with teachers than NT students (Cepero et al., 2023). Interestingly, scores on a language assessment were a poorer predictor of CTCs for autistic children than NT children. The weaker association between assessed language and CTC suggests that for autistic preschoolers, non-linguistic factors, such as differences in social communication or affect, may play a larger role in supporting opportunities for conversation with teachers.

### The timing of vocal exchanges

Researchers have used LENA's speech segmentation and diarization algorithms to measure vocal exchanges as an alternative to LENA's CTC measure. For example, researchers used LENA to assess how parents respond to children's communication attempts. Using LENA speech diarization and recordings of NT and autistic children, Warlaumont et al. (2014) identified their speech-like vocalizations that contained any phonemic production (e.g., babbling and speaking words) and non-speech-like vocalizations (e.g., crying) and adults' responses to these two vocalization types. Adults were more responsive to children's speech-like vocalizations, but this association was stronger for NT children than for autistic children. In general, children's vocalizations were more likely to be speech-like if their previous speech-like vocalization had received a response. These findings provide evidence of a social feedback loop that encourages speech development for all children. However, this feedback loop is weaker for autistic children, perhaps because of a developmental history of producing fewer speech-like vocalizations than NT children, reducing adults' contingent responses to them that would encourage more speech-like vocalizations and more contingent responding.

LENA's CTC measure captures sequential vocalizations by children and adults. However, it fails to measure the reciprocal bidirectionality of vocalizations as it does not account for the possibility of chance sequencing (sequential vocalizations occurring by chance rather than in response to the prior vocalization). According to Harbison et al. (2018), reciprocity occurs when children attend to and respond to adults' responses in a bidirectional manner, which is vital for language development. To better measure the feedback loop occurring in back-and-forth conversations, Harbison et al. (2018) created the Reciprocal Vocal Contingency (RVC) model that accounts for chance probability and measures the sequential association between a child's vocal response to the immediately preceding adult response (ChildVoc-AdultVoc-ChildVoc). Results from LENA recordings of autistic preschoolers' vocalizations showed an association between vocal reciprocity (RVC scores) and children's speech-like vocalizations. Autistic children produced fewer speech-like vocalizations than their NT peers and did not attend as well to adults' responses. As a result, they experienced fewer reciprocal vocal exchanges than their NT peers. This association between feedback loops and speech-like vocalizations supports prior findings that parents are more likely to respond to children when they make speech-like sounds and that in turn, children are more likely to attend and respond to parents' responses.

### Automated measurement of speech characteristics

Atypical speech characteristics such as high-pitched cries and low phoneme count within utterances are associated with autism spectrum disorder (ASD). A meta-analysis of studies across the lifespan demonstrated that autistic individuals tend to demonstrate larger mean, range, and variability of fundamental frequency (perceived pitch) than their NT peers (Asghari et al., 2021). Automated measures allow us to capture vocalization features such as pitch and frequency. When used with LENA recordings, other automated measurement tools like Sphinx-4 and PRAAT can identify and analyze these fine-grained aspects of speech quality in vocalizations. Moffitt et al. (2022) used Sphinx-4 to estimate the number of phonemes (consonant and vowel sounds) and PRAAT to quantify the fundamental frequency of vocalizations during clinical diagnostic observations. Children whose vocalizations contained fewer phonemes and higher pitched cries and speech-like vocalizations were rated by clinicians as exhibiting higher rates of restricted and repetitive behaviors (RRB). This association between vocalization features and clinician-rated RRB scores demonstrates a relationship between fine-grained aspects of speech and ASD symptom severity.

### Vocal interactions and social networks

Social network analysis enables an understanding of how speech and language are shared between individuals. Social network analyses suggest that autistic children are on the periphery of their classroom networks (Locke et al., 2013). Traditionally, this work relied on subjective teacher- and child-reports of friendships. Combining automated measures of location (*via* ultra-wideband radiofrequency identification (RFID) systems like Ubisense) and vocalizations (LENA) allowed researchers to construct *objective* social networks to understand the relative strength of ties between social partners in inclusive preschools (Fasano et al., 2021). Classroom networks were created through data from child-worn LENA recorders and Ubisense RFID tags. In these networks, children (nodes) were connected to one another by ties indexing the summed rate of speech shared between each peer dyad. A child's degree centrality is the sum of all their ties. On average, autistic preschoolers were less central to their classroom networks than NT preschoolers. Across both groups, children that were more central to the network had higher scores on a standardized language assessment than those who were less central, suggesting peer talk may support children's developing language abilities. Using these automated technologies reduced the subjectivity of traditional teacher- and child-report measures of social ties and the time needed to collect measurements, allowing assessment of network changes over the school year.

## Limitations of automated measures

Despite the advantages automated measurement systems afford, there are limitations. For instance, as LENA is an audio recorder, it cannot capture non-verbal communication. Additionally, LENA by itself cannot distinguish between child-directed and overheard speech. Furthermore, reliability comparisons between LENA algorithms and human coders have yielded mixed results. Although several studies have found moderate reliability between LENA and human counts of child vocalizations (CVC) and adult word count (AWC; e.g., Soderstrom and Wittebolle, 2013; Gilkerson et al., 2015; VanDam and Silbert, 2016; Busch et al., 2018; Fasano et al., 2021; Mitsven et al., 2022), a large-scale reliability study found some vocalization types are more likely than others to be misclassified (Cristia et al., 2021; and see e.g., Bulgarelli and Bergelson, 2020). For example, high-pitched female speech can be confused for CVC. Additionally, although LENA and human-coded counts of CVC are highly correlated, LENA seems to systematically underestimate CVC relative to human coders (Marchman et al., 2021). Critically, however, a meta-analysis examining associations between LENA measures and language abilities found moderate effects for both CVC and CTC, and small-to-medium effects for AWC (Wang et al., 2020). These findings suggest that despite occasional errors, LENA-estimated vocalization measures are capturing behaviors meaningful for language development.

Sphinx-4's use is limited by using a model trained on English phonemes. Although alternative language and acoustic models are available, using one model at a time may limit accurate recognition of non-standard dialects or multi-lingual samples (Shmyrev, 2020). The fine-grained features of speech measured by PRAAT can be adjusted by researchers based on their research question (e.g., studies focusing on children's speech would have higher frequency bands than those focusing on adult speech; Gabrieli et al., 2019), introducing possible error in parameter setting. High-quality audio is also vital to obtain accurate measures and output. Ubisense tracks location to an accuracy of 15–30 cm, allowing room for error (Phebey, 2010). Overall, the decision to use any automated technology depends on the researchers' goals. For example, if the research requires an exact count of vocalizations, hand-coding might be best; if the goal is to compare relative amounts of vocalizations or observe how vocalizations are related to other behaviors especially over long timescales or across multiple children, automated measures may be preferable (cf. Marchman et al., 2021).

## Clinical implications

The landscape of automated measurement is shifting rapidly and has implications for clinical practice. In particular, automated measurement holds the potential to transform screening methods for ASD (Dawson and Sapiro, 2019). For example, given that automated vocalization measures are associated with clinician-rated ASD symptom severity (Moffitt et al., 2022) and diagnostic group differences in vocalization pitch are found across the lifespan (Asghari et al., 2021), future screening methods could use these measures to supplement clinician ratings and parent reports of behavior. Automated measures could also be used to monitor behavioral changes associated with parent or clinician implemented interventions. Objective measurement of changes in behavior or symptom severity associated with interventions would enable researchers to measure their efficacy and allow clinicians to make necessary adjustments to best serve individual children. Current work assessing ASD symptoms is typically siloed into observations within home, school, or clinic contexts. Observations of children across contexts are necessary to understand whether cross-context prediction of behaviors is possible or if multi-context observation is necessary to ascertain a clear clinical picture.

## Future directions

Researchers are beginning to use automated technologies to capture non-verbal behaviors such as facial expressions or gestures. As these actions may give insight into ASD symptoms and aid in screening and diagnosis, advancement of automated measurement of nonverbal behaviors should continue to be a focus of research related to ASD. Siddiqui et al. (2021) pilot proof-of-concept study utilized wrist-worn sensors to automatically identify gestures of autistic children (i.e., reaching vs. pointing). Additionally, automated measures can detect behaviors that indicate children's understanding of other's language. For example, Campbell et al. (2019) used computer vision to demonstrate that autistic toddlers were less likely to turn their head to respond to their name, and their responses were a full second slower than NT children when they did. Gaining insight into non-verbal behaviors would be especially useful in improving our understanding of minimally verbal children. Research on non-verbal behaviors would give researchers more insight into how people express themselves irrespective of verbal capabilities.

## Author contributions

EL, DM, and LP conceived of the paper idea. EL wrote the first draft of the manuscript. DM and LP obtained funding to support this work. All authors edited the manuscript. All authors contributed to the article and approved the submitted version.

## Footnotes

^1^In this article, we chose to use identity-first language to refer to different groups due to a growing preference for identity first language in the verbal autistic community. However, we acknowledge that there is still much debate about person- or identity- first language in the field and that individuals have the right to choose how they want to be referred to.

## References

[B1] Asghari S. Z. Farashi S. Bashirian S. Jenabi E. (2021). Distinctive prosodic features of people with autism spectrum disorder: A systematic review and meta-analysis study. Sci. Rep. 11, 23093. 10.1038/s41598-021-02487-6 34845298 PMC8630064

[B2] Bulgarelli F. Bergelson E. (2020). Look who's talking: A comparison of automated and human-generated speaker tags in naturalistic day-long recordings. Behav. Res. Methods 52, 641–653. 10.3758/s13428-019-01265-7 31342467 PMC6980911

[B3] Busch T. Sangen A. Vanpoucke F. van Wieringen A. (2018). Correlation and agreement between Language ENvironment Analysis (LENA™) and manual transcription for Dutch natural language recordings. Behav. Res. Methods 50, 1921–1932. 10.3758/s13428-017-0960-0 28936690

[B4] Campbell K. Carpenter K. L. H. Hashemi J. Espinosa S. Marsan S. Schaich Borg J.. (2019). Computer vision analysis captures atypical attention in toddlers with autism. Autism 23, 619–628. 10.1177/1362361318766247 29595333 PMC6119515

[B5] Cepero C. E. Leland E. Fasano R. M. Messinger D. S. Perry L. K. (2023). Language abilities and conversational turn-taking with teachers among preschoolers with and without autism spectrum disorder, in Poster to Be Presented at the 40th Biennial Meeting of the Society for Research in Child Development. Salt Lake City, UT.

[B6] Chenausky K. V. Maffei M. Tager-Flusberg H. Green J. R. (2022). Review of methods for conducting speech research with minimally verbal individuals with autism spectrum disorder. Augment. Alternat. Commun. 2022, 1–12. 10.1080/07434618.2022.2120071 36345836 PMC10364318

[B7] Cristia A. Lavechin M. Scaff C. Soderstrom M. Rowland C. Räsänen. (2021). A thorough evaluation of the Language Environment Analysis (LENA) system. Behav. Res. Methods. 53, 467–486. 10.3758/s13428-020-01393-5 32728916 PMC7855224

[B8] Dawson G. Sapiro G. (2019). Potential for digital behavioral measurement tools to transform the detection and diagnosis of autism spectrum disorder. J. Am. Med. Assoc. Pediatr. 173, 305–306. 10.1001/jamapediatrics.2018.5269 30715131 PMC7112503

[B9] Donnelly S. Kidd E. (2021). The longitudinal relationship between conversational turn-taking and vocabulary growth in early language development. Child Dev. 92, 609–625. 10.1111/cdev.13511 33547640

[B10] Fasano R. M. Perry L. K. Zhang Y. Vitale L. Wang J. Song C.. (2021). A granular perspective on inclusion: Objectively measured interactions of preschoolers with and without autism. Aut. Res. 14, 1658–1669. 10.1002/aur.2526 33938641

[B11] Gabrieli G. Leck W. Q. Bizzego A. Esposito G. (2019). Are PRAAT's default settings optimal for infant cry analysis?, in Proceedings of the 17th Linux Audio Conference (LAC-19) (Stanford, CA: Stanford University), 1–6.

[B12] Gilkerson J. Richards J. A. Warren S. F. Oller D. K. Russo R. Vohr B. (2018). Language experience in the second year of life and language outcomes in late childhood. Pediatrics 142, e20174276. 10.1542/peds.2017-4276 30201624 PMC6192025

[B13] Gilkerson J. Zhang Y. Xu D. Richards J. A. Xu X. Jiang F.. (2015). Evaluating language environment analysis system performance for Chinese: A pilot study in Shanghai. J. Speech Lang. Hear. Res. 58, 445–452. 10.1044/2015_JSLHR-L-14-0014 25614978

[B14] Harbison A. L. Woynaroski T. G. Tapp J. Wade J. W. Warlaumont A. S. Yoder P. J. (2018). A new measure of child vocal reciprocity in children with autism spectrum disorder: Child vocal reciprocity. Aut. Res. 11, 903–915. 10.1002/aur.1942 29509308 PMC6026061

[B15] Locke J. Kasari C. Rotheram-Fuller E. Kretzmann M. Jacobs J. (2013). Social network changes over the school year among elementary school-aged children with and without an autism spectrum disorder. School Mental Health 5, 38–47. 10.1007/s12310-012-9092-y

[B16] Marchman V. A. Weisleder A. Hurtado N. Fernald A. (2021). Accuracy of the Language Environment Analyses (LENATM) system for estimating child and adult speech in laboratory settings. J. Child Lang. 48, 605–620. 10.1017/S0305000920000380 32690113 PMC8178803

[B17] Mitsven S. G. Perry L. K. Tao Y. Elbaum B. E. Johnson N. F. Messinger D. S. (2022). Objectively measured teacher and preschooler vocalizations: Phonemic diversity is associated with language abilities. Dev. Sci. 25. 10.1111/desc.13177 34592032 PMC8847312

[B18] Moffitt J. M. Ahn Y. A. Custode S. Tao Y. Mathew E. Parlade M.. (2022). Objective measurement of vocalizations in the assessment of autism spectrum disorder symptoms in preschool age children. Aut. Res. 15, 1665–1674. 10.1002/aur.2731 35466527

[B19] Phebey T. (2010). The Ubisense assembly control solution for BMW solution for BMW, in Proceedings of RFID Journal Europe Live (Darnstadt).

[B20] Romeo R. R. Choi B. Gabard-Durnam L. J. Wilkinson C. L. Levin A. R. Rowe M. L.. (2022). Parental language input predicts neuroscillatory patterns associated with language development in toddlers at risk of autism. J. Aut. Dev. Disord. 52, 2717–2731. 10.1007/s10803-021-05024-6 34185234 PMC9594983

[B21] Romeo R. R. Leonard J. A. Grotzinger H. M. Robinson S. T. Takada M. E. Mackey A. P.. (2021). Neuroplasticity associated with changes in conversational turn-taking following a family-based intervention. Dev. Cogn. Neurosci. 49, 100967. 10.1016/j.dcn.2021.100967 34052580 PMC8175277

[B22] Shmyrev N. (2020). Frequently Asked Questions (FAQ). CMUSphinx Open Source Speech Recognition. Available online at: https://cmusphinx.github.io/wiki/faq/ (accessed March 1, 2023).

[B23] Siddiqui U. A. Ullah F. Iqbal A. Khan A. Ullah R. Paracha S.. (2021). Wearable-sensors-based platform for gesture recognition of autism spectrum disorder children using machine learning algorithms. Sensors 21, 3319. 10.3390/s21103319 34064750 PMC8150794

[B24] Soderstrom M. Wittebolle K. (2013). When do caregivers talk? The influences of activity and time of day on caregiver speech and child vocalizations in two childcare environments. PLoS ONE 8, e80646. 10.1371/journal.pone.0080646 24260443 PMC3832484

[B25] VanDam M. Silbert N. H. (2016). Fidelity of automatic speech processing for adult and child talker classifications. PLoS ONE 11, e0160588. 10.1371/journal.pone.0160588 27529813 PMC4986949

[B26] Wang Y. Williams R. Dilley L. Houston D. M. (2020). A meta-analysis of the predictability of LENA™ automated measures for child language development. Dev. Rev. 57, 100921. 10.1016/j.dr.2020.100921 32632339 PMC7337141

[B27] Warlaumont A. S. Richards J. A. Gilkerson J. Oller D. K. (2014). A social feedback loop for speech development and its reduction in autism. Psychol. Sci. 25, 1314–1324. 10.1177/0956797614531023 24840717 PMC4237681

[B28] Warren S. F. Gilkerson J. Richards J. A. Oller D. K. Xu D. Yapanel U.. (2010). What automated vocal analysis reveals about the vocal production and language learning environment of young children with autism. J. Aut. Dev. Disord. 40, 555–569. 10.1007/s10803-009-0902-5 19936907

